# Food Acquisition during the COVID-19 Lockdown and Its Associations with the Physical–Digital Integrated Community Food Environment: A Case Study of Nanjing, China

**DOI:** 10.3390/ijerph19137993

**Published:** 2022-06-29

**Authors:** Zhongyu He, Weijie Pan

**Affiliations:** School of Architecture and Urban Planning, Nanjing University, Nanjing 210093, China; mf21360089@smail.nju.edu.cn

**Keywords:** food acquisition, community food environment, digital food environment, COVID-19, online shopping, accessibility

## Abstract

The COVID-19 pandemic and measures such as lockdowns affect food access, dietary choices, and food security. We conducted an online survey among 517 respondents during early 2020 in Nanjing, China to explore respondents’ food acquisition behaviors before and during the pandemic and associations with the community food environment. Using geographic analysis and binary logistic models, we revealed that despite inconvenience regarding food acquisition, no food security issues occurred during lockdown in Nanjing. The pandemic changed the access and frequency of obtaining food; meanwhile, pre-pandemic habits had a strong impact on food acquisition behavior. Online and in-store food acquisition showed a substitution relationship, with online food access playing a crucial role in food acquisition. Physical and digit food outlets are highly integrated in Chinese urban communities, and both objectively measured and perceived accessibility of these food outlets had a significant association with the food acquisition methods and transportation mode chosen by people during this public health crisis.

## 1. Introduction

The emergence of the novel coronavirus SARS-CoV-2 and subsequent global COVID-19 pandemic has become an unprecedented public health emergency and has led to worldwide economic and social crises [1]. Supply chain disruptions due to the pandemic are affecting consumer food environments that, coupled with COVID-19 mitigation measures such as lockdowns, influence food access, dietary choices, and food security [2]. Concerns about food shortages cause excessive demand and panic buying [3]. Social distancing policies were issued, and restaurants were mandated to close in countries worldwide. As a result, people had to cook at home more often [4]. People’s mobility was limited in many places, which shifted the transportation preferences from public and shared transport to walking or using private vehicles [5]. To reduce the risk of exposure to the virus, many consumers shifted to online food shopping [6]. Regarding dietary choices, one study on parents’ feeding practices showed dramatically increased consumption of high-calorie, non-perishable processed food during the COVID-19 pandemic, which increased the negative effects of the pandemic on children’s obesity risk [7]. In the United States (U.S.), scholars reported that the pandemic had disproportionately affected food access for groups that already had higher rates of food insecurity [8], and food insecurity emerged among adults who experienced many barriers to maintaining healthy eating habits during the pandemic [9]. 

Coping with strains on food systems during a public health emergency is of utmost importance because the demand for food is ongoing and closely related to people’s health status. Existing literature provides some evidence regarding effects of the pandemic on consumer behavior, yet little is known about how these effects are associated with the local food environment. In addition, online grocery shopping has witnessed stable growth in the last decade, and a significant increase of retailing occurred online is observed during the pandemic crisis [10]; however, few studies have explored how the widespread use of digital technology impact the way people obtain food during COVID-19 and its relationship with the traditional in-store food shopping. We conducted an empirical study in Nanjing, China to answer the following two questions: (1) How did people’s food acquisition behavior change during the COVID-19 lockdown, compared with that before the pandemic? (2) How is this behavior associated with the objectively measured and subjectively perceived community food environment (CFE), by taking into account of various physical and digital food outlets?

## 2. Literature Review

The food environment can be conceptualized as including all places and pathways through which people acquire and/or consume food and the various characteristics of those environments that influence food choices [11]. Turner et al. [12] proposed an eight-dimension framework of the food environment. Healthy dietary behavior is more likely to occur in a supportive environment with easily accessible healthy food. Recently, influences of the food environment on dietary behavior and human health have become a global concern [13]. 

The food environment can be categorized into the community food environment, organizational food environment, consumer food environment, and the information environment [14]. Several different methods have been applied among studies in this field, including geographic analysis, food supply analysis, menu analysis, and nutrient analysis [15]. Among these methods, geographic analysis, used to measure accessibility of the food environment, is the most frequently used. From the demand (consumer) perspective, accessibility can be measured using the frequency [16], proximity [17], or presence [18] of food outlets. More complex models such as E2SFCA (enhanced two-step floating catchment area) (The method is a special case of the gravity model; it conducts a two-step search from the locations of demand and supply, respectively, and it improves the accessibility measurement by considering the capacity of the supply end.) [19] are adopted when both demand and supply sides must be taken in account. Using the aforementioned techniques, various types of food outlet have been investigated, including fast food restaurants [20], convenience stores [21], grocery stores [22], supermarkets [23], and full-service restaurants [24]. The results have been mixed, with fast food restaurants and convenience stores hypothesized to contribute to the consumption of unhealthy food and obesity and supermarkets hypothesized to increase the consumption of fruits and vegetables and to therefore promote a healthy weight [25]. For example, one study in China found that community exposure to Western fast food was positively associated with subsequent increased body weight [26]. Similarly, in the Netherlands, exposure to fast food outlets was found to be positively associated with diabetes prevalence, although the effect estimates were small [27]. Another study in the U.S. concluded that supermarkets were positively related to a lower prevalence of obesity [28].

In recent years, the digitalization of food environments has become a central issue in public health and reshaping food availability, acquisition, and consumption [29]. This trend has the potential to increase the availability of a wider range of foods through online grocery shopping [30]. At the same time, digital and physical food environments are interconnected and influence one another. For example, physical food vendors are undergoing digital enhancement [31]. Shi et al. [32] found that online shopping frequency is affected by the built environment via the mediating role of attitudes about shopping. Huang et al. [33] argued that Food-related tweets can help characterize the neighborhood food environment, which in turn are linked with community levels of obesity and hypertension. It is therefore necessary to integrate the digital food environment in consideration of the CFE.

## 3. Characteristics of the CFE in Chinese Cities

Owing to the different food culture, diet, and consumer preferences, the CFE in Chinese cities is unique in comparison with that in Western contexts. For example, although fast food restaurants in the West are generally considered outlets of unhealthy food, Chinese-style fast food may have different characteristics. Zhou et al. [34] found that Chinese-style fast food reduced the risk of overweight and obesity in children. Farmer’s markets are important sources of fresh food in China. These markets are planned and constructed by the government on a population basis and are usually located within a walkable distance from local communities, which differs from farmer’s market in Western counties. Another characteristic of the CFE in China is the popularity of online-to-offline (O2O) food delivery services. More than one-fifth of all people in China have become users of O2O food delivery services [35]. Owing to the well-developed logistics system and low cost of delivery, nearly all types of food, from prepared dishes to fresh vegetables and fruits or frozen food products, can be delivered at the same or at a lower price than buying in the store.

Table 1 presents a summary of the major types of food outlets in communities throughout Chinese cities. The online–offline integrated (OOI) store is a new mode of retail first proposed by Alibaba that emerged in China in 2016. OOI aims to closely integrate online platforms and offline stores with modern logistics using big data and artificial intelligence technologies [36]. As a food outlet, an OOI store sells fresh food and provides onsite cooking services. Consumers can order fresh or prepared food online and have it delivered as well. Warehouse-based shopping (WBS) applications (apps) have also appeared in recent years. Compared with traditional online shopping (TOS) websites with central storage, WBS involves a network of warehouses aimed to guarantee prompt delivery. The aforementioned three types of food outlet all have their own logistics system. Some restaurants, supermarkets, and convenience or grocery stores provide delivery service in cooperation with a third-party app such as Meituan or Elema. All these online food outlets provide delivery services via nearby physical stores or warehouses, within a typical delivery radius of 3 km. The exception is the TOS website, which has no distance limit.

## 4. Methodology

### 4.1. Study Area and Data Collection

This empirical study was carried out in Nanjing, the capital city of China’s Jiangsu Province. By the end of 2021, the city had a population of 9.42 million with a gross domestic product per capita of approximately 27,200 USD. The SARS-CoV-2 virus was first reported in Wuhan at the end of 2019, and the city was locked down from 23 January 2020. By February 2020, all 31 provinces of mainland China had reported confirmed cases of COVID-19 and strict policies were issued to prevent spread of the virus, including social distancing, mandatory mask wearing, and body temperature monitoring in public venues. People were encouraged to work at home, and most commercial businesses were closed, except for food stores. People’s mobility was limited, and visitors were not allowed to enter gated communities. During this first wave of the pandemic, Nanjing reported a total of approximately 100 COVID-19 cases. Our investigation took place from 21 to 27 March 2020, when the pandemic was mostly under control. We designed the questionnaire and distributed it using an online survey service (www.wjx.cn (accessed on 10 March 2020)). The respondents were randomly chosen by receiving invitation links; however, they should meet the following screening criterions: he/she should be (1) an adult resident living in Nanjing for at least 6 months; (2) residing in the same place before and during the pandemic lockdown; and (3) not infected by COVID-19 or a close contact of anyone infected. Qualified respondents were provided an instruction of the survey as well as our privacy policy and contact of the survey instructor; upon their agreement to participate, they can proceed to answer the questions. Finally, 517 valid questionnaires were collected from 644 respondents.

Respondents were asked about their frequency, transportation mode, and travel (delivery) time to different in-store and online food outlets before and during the pandemic. Using a 5-point Likert scale, we also queried perceptions regarding the accessibility, availability, and affordability of food options during the pandemic lockdown period (25 January to 29 February 2020) as well as respondents’ evaluation of the effect of the pandemic on their diet. We collected demographic information, as well as the home address and body mass index (BMI) of respondents at the end of the questionnaire. Point-of-interest data of different food outlets and the road network were crawled from the Internet. By March 2020, we identified 36,872 restaurants (including full-service and fast food), bakeries, or beverage stores, among which 14,141 provided food delivery service; 321 supermarkets, 374 farmer’s markets, 17 OOI stores, 34 WBS warehouses, and 9465 convenience or grocery stores (6902 of which provided online shopping service) in Nanjing. Grocery stores were included only when their name clearly indicated that they sold food; this would lead to underestimation of the number of grocery stores that should have been included. 

### 4.2. Methodology and Hypothesis

In this study, we defined food acquisition behavior as the access, frequency, transportation mode, and time spent for people to obtain fresh and/or prepared food. Food access can be divided into buying from food outlets and self-cooking, eating out in restaurants, or ordering in from restaurants. Food buying further includes buying online and in-store buying. For the CFE, we considered the 3A dimensions, namely, the accessibility, availability, and affordability of food options. Owing to the large size of the data set, we measured the latter two only using respondents’ perceptions according to the survey; accessibility was measured both objectively and subjectively.

We first conducted a geographic analysis to visualize the distribution of the CFE in Nanjing using the Kernel density tool in ArcMap. Then we measured the accessibility of the CFE. As mentioned in the previous section, there are several ways to measure accessibility; here, we chose density. All food outlets and respondents’ home addresses were located on the city map according to their coordinates. For each home address, a 1-km buffer (the distance covered on an approximately 15-min walk) and a 3-km buffer (maximum delivery distance) were generated and the number of different food outlets within the buffer areas were calculated. We ran several binary logistics models using IBM SPSS 22 (IBM Corp., Armonk, NY, USA) to analyze the associations between dietary behavior and the CFE during the pandemic. According to the findings of previous studies, we put forth the following hypotheses:

**Hypothesis** **1.** *Food acquisition behavior during the COVID-19 pandemic is associated with the way people obtain food before the pandemic*.

**Hypothesis** **2.** *There is a substitution effect between online and in-store food shopping*.

**Hypothesis** **3.** *The accessibility of the CFE has a positive association with food acquisition*.

**Hypothesis** **4.** *Food acquisition behavior is influenced by consumers’ perceptions about the CFE*.

**Hypothesis** **5.** *The accessibility of the CFE is positively associated with the walking mode of transport but negatively associated with driving mode for food shopping*.

## 5. Results

### 5.1. Descriptive Analysis

Most survey respondents were in the age groups 31–40 (36.28%) and 19–30 (25.14%) years; only 7.30% of respondents were aged 51 years and above. Older people were underrepresented in this study because the survey was carried out online. In total, 37.3% of respondents were overweight (BMI > 24 kg/m^2^), and 44.9% reported an increase in body weight during the lockdown. In total, 15.9% of respondents had confirmed cases of COVID-19 infection in or near their community.

Table 2 shows the changes in food acquisition behavior before and during the pandemic. The pandemic largely discouraged people from eating out and influenced their food buying behavior but greatly increased the frequency of ordering in. Regarding food shopping access, the most popular food outlets were farmer’s markets and supermarkets before the pandemic; this changed to online shopping during the pandemic. Online group purchases organized within the community also increased during the pandemic and served as an important way to obtain fresh food. Transportation modes and travel times did not change much except that people used less public transit. Delivery time for both fresh food and prepared meals was longer during the pandemic. The most frequently used online outlets for fresh food were TOS websites both before and during the pandemic, followed by OOI stores. People tended to order a greater variety of prepared meals during than before the pandemic.

In terms of perceptions about the CFE during the pandemic compared with previously, 82.53% of respondents agreed or strongly agreed that in-store food shopping was less accessible whereas this proportion was only 45.11% for online food shopping. Between 53.55% and 69.87% of respondents believed that food was less available in different food outlets. On average, approximately 60% of respondents felt that food was less affordable, especially that from a supermarket or farmer’s market.

**Figure 1:**
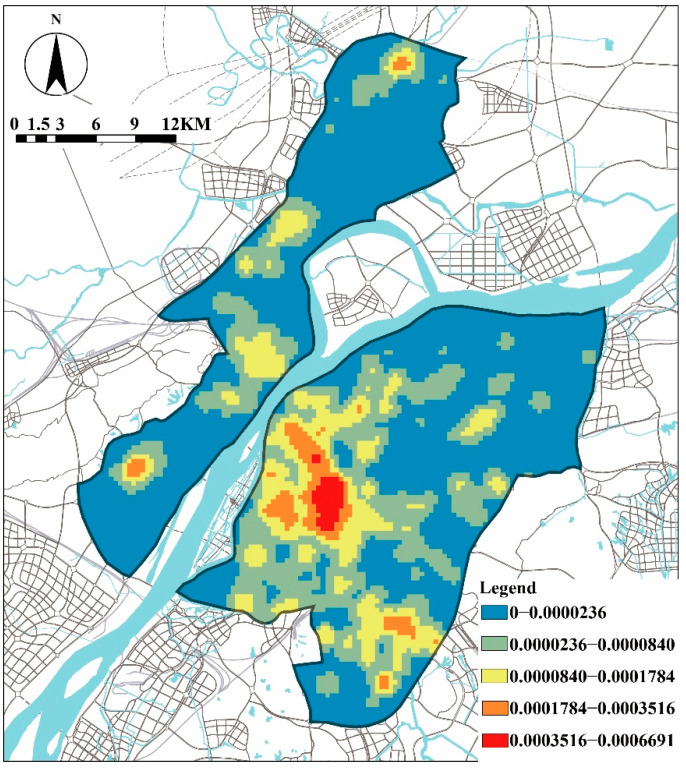
Density of food outlets in Nanjing.

**Figure 2:**
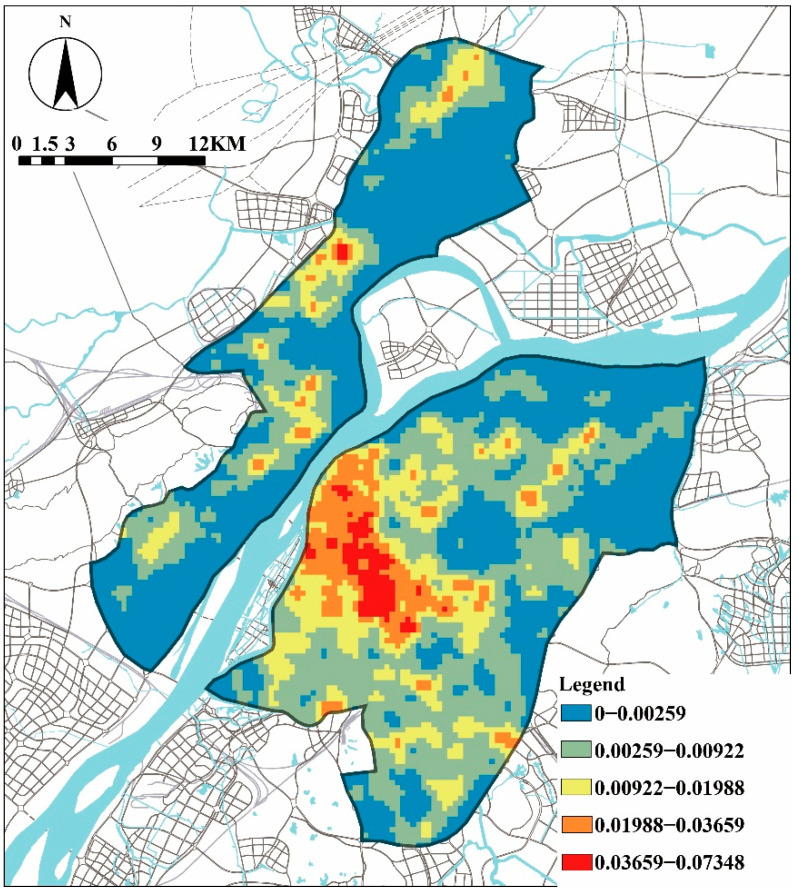
Density of the population in Nanjing.

Table 3 shows the average, maximum, and minimum number and the standard deviation for each food outlet within the aforementioned buffers for the 517 respondents. The density of different food outlets was highly correlated among them (correlation coefficient 0.375–0.965, *p* < 0.000), and this also had a positive relationship with the population density of the city (Figure 1 and Figure 2).

### 5.2. Statistical Analysis

The survey included 14 questions on respondents’ perceptions about the CFE during the pandemic, scored using a 5-point Likert scale (1: strongly disagree to 5: strongly agree). To reduce the dimensionality of these 14 variables, we carried out principal component analysis (PCA). The KOM value of the variables was 0.774, indicating the validity of PCA. Table 4 shows the rotated component matrix: Component 1 reflects perceptions about food affordability; Component 2 reflects perceptions about food availability; and Components 3 and 4 reflect perceptions about the accessibility to food in-store and online, respectively. Safety concerns (namely, the risk of being infected when going out to shop for food or receiving a food delivery) are included in the accessibility components, based on the results of PCA.

We investigated two transportation modes (walking and driving) for food shopping before and during the pandemic and their correlation with the eight objectively measured CFE items in Table 3 and the four perceived CFE items in Table 4. As shown in Table 5, both modes were significantly related to objectively measured CFE items but with opposite effects. Among the four dimensions of perceived CFE during the pandemic, only accessibility was related to the choice of transportation mode. The correlation coefficients were larger during than before the pandemic for both modes. Walking was more closely related to the digital CFE, and driving was more related to the physical CFE. Monthly family income was also positively correlated with the digital CFE. However, no significant relationship was found between BMI or overweight status and the CFE.

Table 6 shows results of the logistic models. The dependent variable was whether respondents engaged in one of the three food acquisition behaviors during the pandemic (eating out was excluded because more than 95% of respondents never ate out during the lockdown period). There were six categories of independent variable: whether respondents engaged in other food acquisition behaviors during the pandemic and the four food acquisition behaviors before the pandemic; accessibility of the physical CFE; accessibility of the digital CFE, the perceived CFE, and personal attributes. Only statistically significant variables were included in the final models. Since the eight objectively measured CFE variables were highly correlated, only the most significant one among the eight variables was retained to avoid multicollinearity.

Food acquisition behavior before the pandemic was the variable most highly correlated with the same behaviors during the pandemic. For example, respondents who did their shopping online before the pandemic had a 10.282 times greater likelihood of doing their shopping online during the pandemic than those who had never shopped online before the pandemic. On the other hand, during the pandemic, online and in-store shopping behavior had a negative impact on each other. Namely, respondents who shopped in-store were less likely to shop online, as compared with those did not shop in-store, and vice versa. The most relevant objectively measured CFE variable in terms of choosing online food shopping, in-store food shopping, and ordering in was accessibility of a supermarket, OOI store, and online convenience or grocery store, respectively. Regarding the perceived CFE during the pandemic, a 1-point higher score in agreement that food delivery was not as convenient as before the pandemic or that food delivery could increase the chance of COVID-19 infection reduced the likelihood of shopping online by 42.9% and of ordering in by 53.9%. A 1-point higher score regarding concern about the accessibility and safety of in-store food shopping reduced the likelihood of shopping in-store by 18.9%. Food affordability and availability were mostly non-significantly associated with food acquisition behavior. As for personal attributes, younger age and larger family size were associated with a greater likelihood of choosing in-store food shopping. Family income was positively associated with online food shopping and ordering-in behaviors. Respondents who rented rather than owned an apartment or had lower BMI were more likely to shop online; those who had confirmed COVID-19 cases nearby were less likely to order food in.

## 6. Discussion

Using data from an online survey of 517 valid respondents conducted in early 2020, immediately following the first wave of the COVID-19 pandemic in Nanjing, China, we explored respondents’ food acquisition behaviors before and during the lockdown period and their associations with the CFE. Although in general, people relied on online food acquisition more so than in-store shopping during the COVID-19 pandemic lockdown in Nanjing, our regression models showed that the usual preference of acquiring food was still the most significant factor influencing how people obtained food during the COVID-19 crisis. This relation is neglected in some similar studies comparing food shopping before and during the pandemic [37,38], but it is in line with a study by Chen et al. [39] in Wuhan. Our hypothesis 1 was therefore validated. In addition, this finding reveals that compared with external factors (e.g., the pandemic), internal factors (e.g., habit/preference) have a much stronger impact on people’s behavior.

The relationship between online and in-store shopping has been well studied, and four types of relationship have been proposed: substitution, complementarity, neutrality, and modification [40,41]. However, these relationships have seldom been tested for food shopping during a large-scale and long-lasting public health crisis. Our research showed that under such circumstances, online and in-store food acquisition were more likely to substitute each other. We attribute this to the fixed demand for food; especially during the pandemic, people tended to minimize food shopping frequency. Furthermore, we determined that one type of online shopping (such as food buying) not only discouraged in-store shopping but also encouraged another type of online shopping (such as ordering in). Our model also showed that one pair of behaviors, ordering in and eating out, were positively associated, which indicates a complementary relationship. Therefore, hypothesis 2 was partly validated.

Our research supports that the CFE has a minor, positive, but statistically significant effect on dietary behavior, as many previous studies have suggested [42,43]. Different food acquisition behaviors were differently associated with the objectively measured CFE: online food buying was associated with the accessibility of most food outlets, regardless of whether physical or digital. Ordering in was associated with digital food outlets only, and in-store food buying was not associated with most food outlets. We assume that this is because online shopping or ordering in are relatively flexible and more readily available options that will induce more consumption. Owing to safety concerns during the pandemic, people had already minimized going out to shop; therefore, shopping trips that did occur were out of necessity, regardless of whether the food outlets were easily accessible. To verify this assumption, we checked the relationship between in-store shopping and food accessibility before the pandemic and found that a density of five out of the eight food outlets was positively associated with food buying behavior (*p*-value from 0.026 to 0.062), which indicates that normal food shopping was more likely to occur in a CFE with greater accessibility. Another observation is that a digital CFE had an equally strong, if not stronger, association as a physical CFE with food acquisition behavior, especially during the pandemic lockdown. Thus, our hypothesis 3 was proven correct.

We observed that in Chinese urban communities, most digital food outlets possess some form of physical space (e.g., an independent store, a warehouse, or a cooperating store). This coexistence of digital and physical food outlets has its significance in at least two ways: before the pandemic, physical food stores rely on digital channels to expand their business; while during the crisis, digital food stores can provide prompt food delivery based on the offline network. The phenomenon has noteworthy implications for urban planners and policymakers.

The perceived CFE has a stronger effect on food acquisition behaviors than the objectively measured CFE but is only limited to food accessibility; food affordability and availability did not seem to be a concern during the COVID-19 crisis. Furthermore, we found that perceived online and in-store accessibilities have divergent effects. Therefore, hypothesis 4 was partly validated.

The mode of transportation before and during the pandemic did not change significantly, except that peoples’ use of public transit declined dramatically. As we had assumed, better accessibility of food outlets in the community encourages people to walk more and drive less, which increases their level of physical activity and can positively affect their health. Our hypothesis 5 was therefore proven. However, we did not find any significant change in the travel time for food shopping.

Personal attributes were also found to be associated with food acquisition. Younger people and those with a large family tended to do more in-store shopping during the pandemic, and people with higher incomes were more likely to choose online shopping and ordering in. Although 37.3% of respondents were overweight, our analysis did not show any association between BMI and food acquisition behaviors. We attribute this result to the following. First, acquiring food is usually carried out for the whole family and is not necessarily related to the food intake of the person surveyed. Second, food acquisition is not equal to food consumption; people can acquire foods that make up a healthy diet from different food outlets regardless of whether online or in-store.

This paper has several limitations. First, because this survey was carried out online and older people have limited access to the Internet, our sample was not representative of all residents of Nanjing; therefore, our results could be biased. Second, there are a variety of methods to measure accessibility of the CFE, but we only used one measure. Future studies should include other measurements to investigate food acquisition behaviors. Third, we do not know whether the observed change in food acquisition behavior during the COVID-19 pandemic is temporary or whether it will affect future consumption; this is another important topic for future study.

## 7. Conclusions

The findings of the current research make the following contributions. First, our study investigated the effect of both the CFE and a large-scale public health crisis on consumer behaviors regarding food acquisition; previous studies have mostly focused on only one of these factors. Second, our study integrated both physical and digital food outlets in analyzing the CFE within the context of Chinese cities, where online shopping is rapidly developing.

This paper revealed the perspective of local residents in that despite inconveniences in acquiring food brought about by the COVID-19 pandemic, no serious problems in obtaining food occurred. This could be attributed to the effective control of virus spread and the top priority on food security in the Chinese government’s response to COVID-19 [44]. Food acquisition during the pandemic was found to be strongly associated with consumers’ habits or preferences before the pandemic. Online and in-store food acquisition have a substitution relationship. Physical and digital food outlets are highly integrated in Chinese communities, and no matter whether objectively measured or perceived, their accessibility showed a minor but significant association with food acquisition behavior according to our study. This suggests the importance of urban planning in shaping and organizing the built and natural environments in a way that maximizes population health gains [45]. In our study, food access was well correlated with population density; however, we found a positive association between the density of community food outlets and the household income of community members, which implies possible inequities in the CFE of Chinese cities.

## Figures and Tables

**Table 1:** Food outlet types in the community food environment of Chinese cities.

Food Outlet	In-Store	Online	Delivery Time
Restaurant/bakery/beverage store	Yes	Partly yes	0.5–1 h
Supermarket	Yes	Mostly yes	0.5–1 h
Farmer’s market	Yes	No	-
Convenience/grocery store	Yes	Partly yes	0.5–1 h
Online-offline integrated (OOI) store	Yes	Yes	0.5–1 h
Warehouse based shopping (WBS) app	No	Yes	0.5–1 h
Traditional online shopping (TOS) website	No	Yes	Several days

**Table 2:** Change in food acquisition behavior before and during the pandemic.

Most frequent food access	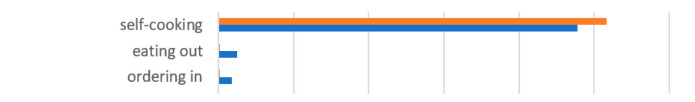
Least frequent food access	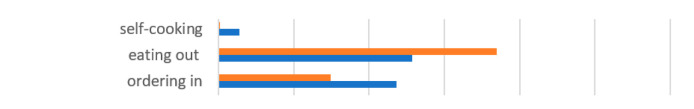
Food shopping access	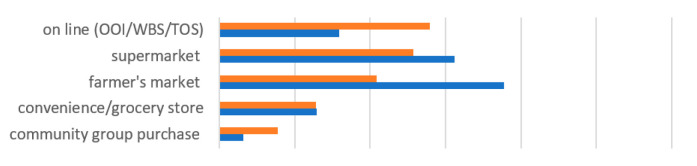
Frequency of eating out per week	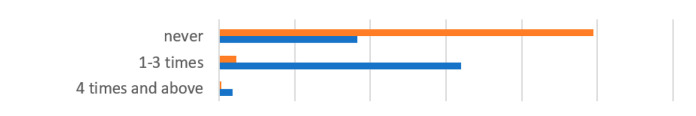
Frequency of ordering in per week	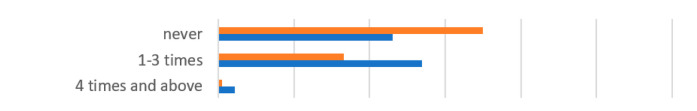
Frequency of food shopping per week	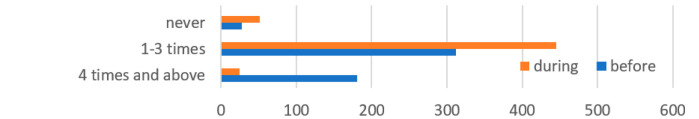

**Table 3:** Number of different food outlets in sampled communities.

Food Outlet		Avg.	Max.	Min.	Std.
Physical CFE (1 km Buffer)	(1) Restaurant/bakery/beverage store	258.94	1647	0	300.04
	(2) Supermarket	1.99	13	0	2.41
	(3) Farmer’s market	10.14	39	0	8.23
	(4) Convenience/grocery store	49.12	193	0	40.83
Digital CFE (3 km Buffer)	(5) Online-offline integrated (OOI) store	1.13	7	0	1.79
	(6) Warehouse based shopping (WBS) app	1.71	8	0	1.68
	(7) Restaurant etc. with delivery service	764.43	2911	0	731.62
	(8) Convenience/grocery store with delivery service	309.47	959	0	249

**Table 4:** Results of principal component analysis regarding perceptions of the CFE during the pandemic.

Statement in the Questionnaire	Component 1	Component 2	Component 3	Component 4
Food price online increased dramatically	0.862	0.137	0.043	0.126
Food price in the supermarket, farmer’s market increased dramatically	0.809	0.220	0.036	−0.007
Order-in price increased dramatically	0.778	0.127	0.029	0.134
Food options online decreased dramatically	0.190	0.734	0.003	0.173
Food options for order-in decreased dramatically	0.041	0.677	0.075	0.170
Food options in the supermarket, farmer’s market decreased dramatically	0.275	0.656	0.085	0.028
Going out for shopping or eating increased the chance of infection	−0.030	0.046	0.718	0.162
My community had strict restrictions on entering or going out	−0.060	0.066	0.704	−0.006
My family had a healthier diet	0.097	−0.250	0.535	0.268
The pandemic brought much trouble on diet for my family	0.111	0.348	0.491	−0.095
Going out for food shopping was not as convenient as before	0.196	0.353	0.449	0.111
Online food shopping was not as convenient as before	0.026	0.260	−0.016	0.781
Food delivery or ordering-in increased the chance of infection	0.135	−0.076	0.317	0.636
Ordering-in was not as convenient as before	0.140	0.414	0.068	0.600

**Table 5:** Correlation between transportation mode, family income, and the community food environment.

Mode	Restaurant/Bakery/Beverage Store	Supermarket	Farmer’s Market	Convenience/Grocery Store	OOI Store	WBS App Warehouse	Restaurant etc. with Delivery Service	Convenience/Grocery Store (Online)	Accessibility
Walk-during	0.106 **	0.126 ***	0.085 *	0.126 ***	0.140 ***	0.143 ***	0.085 *	0.145 ***	−0.105 **
Drive-during	−0.161 ***	−0.122 ***	−0.096 **	−0.161 ***	−0.136 ***	−0.137 ***	−0.079 *	−0.115 **	0.195 ***
Walk-before	0.062	0.158 ***	0.033	0.112 **	0.100 **	0.123 ***	0.104 **	0.089 **	-
Drive-before	−0.112 **	−0.105 **	−0.070	−0.112 **	−0.081 *	−0.103 **	−0.088 *	−0.078 *	-
Family income	0.025	0.086 **	0.013	0.066	0.085 **	0.104 **	0.103 **	0.081 *	-

*** *p*-value < 0.01, ** *p*-value < 0.05, * *p*-value < 0.1.

**Table 6:** Results of logistic regression analysis.

	Dependent Variable	Online	In-Store	Ordering In
Independent Variable		OR (SE)	OR (SE)	OR (SE)
Food acquisition before the pandemic	Online shopping (0: no; 1: yes)	11.282 (0.280) ***		
	In-store shopping (0: no; 1: yes)		10.651 (0.312) ***	
	Eating out (0: no; 1: yes)			
	Ordering in (0: no; 1: yes)			20.870 (0.329) ***
Food acquisition during the pandemic	Online shopping (0: no; 1: yes)		0.349 (0.208) ***	
	In-store shopping (0: no; 1: yes)	0.451 (0.217) ***		
	Eating out (0: no; 1: yes)		1.696 (0.307) *	2.195 (0.308) **
	Ordering in (0: no; 1: yes)	1.752 (0.231) **		
Physical CFE density	Restaurant/bakery/drinks store			
	Supermarket	1.120 (0.045) **		
	Farmer’s market			
	Convenience/grocery store			
Digital CFE density	OOI store		1.090 (0.057) *	
	WBS app warehouse			
	Restaurant etc. with delivery service			
	Convenience/grocery store (online)			1.01 (0.006) *
Perception about CFE during the pandemic	Food affordability			
	Food availability			1.78 (0.235) **
	In-store food accessibility and safety		0.811 (0.123) *	
	Online food accessibility and safety	0.571 (0.223) **		0.461 (0.249) ***
Personal attribute	Age (ordered)		0.744 (0.156) ***	
	Family size		1.378 (0.156) **	
	Income (ordered)	1.544 (0.102) ***		1.229 (0.107) *
	Housing tenure (0: rental; 1: private)	0.584 (0.283) *		
	BMI	0.936 (0.033) **		
	Confirmed case nearby (0: no; 1: yes)			0.521 (0.302) **
Constant		8.069(1.030) **	−1.627(0.921) *	0.025 (0.901) ***
No. of observations: 517	Cox and Snell R^2^	0.288	0.220	0.306
	Nagelkerke R^2^	0.385	0.297	0.427

*** *p*-value < 0.01, ** *p*-value < 0.05, * *p*-value < 0.1; CFE, community food environment; OOI, online–offline integrated; WBS, warehouse-based shopping; OR, odds ratio; SE, standard error. The independent variables are continuous if not specified in the table.

## Data Availability

Not applicable.

## References

[1] Kansiime M.K., Tambo J.A., Mugambi I., Bundi M., Kara A., Owuor C. (2021). COVID-19 implications on household income and food security in Kenya and Uganda: Findings from a rapid assessment. World Dev..

[2] Ahmed S., Downs S.M., Yang C., Chunlin L., Broek N.T., Ghosh-Jerath S. (2020). Rapid tool based on a food environment typology framework for evaluating effects of the COVID-19 pandemic on food system resilience. Food Secur..

[3] Hall C.M., Fieger P., Prayag G., Dyason D. (2021). Panic Buying and Consumption Displacement during COVID-19: Evidence from New Zealand. Economies.

[4] Scarmozzion F., Visioli F. (2020). COVID-19 and the subsequent lockdown modified dietary habits of almost half the population in an Italian sample. Foods.

[5] Braut B., Migheli M., Truant E. (2022). Household mobility in food purchasing during COVID-19 lockdown: Evidence from Torino, Italy. Cities.

[6] Ben Hassen T., El Bilali H., Allahyari M.S. (2020). Impact of COVID-19 on food behavior and consumption in Qatar. Sustainability.

[7] Adams E.L., Caccavale L.J., Smith D., Bean M.K. (2020). Food insecurity, the home food environment, and parent feeding practices in the era of COVID-19. Obesity.

[8] Leone L.A., Fleischhacker S., Anderson S.B., Harper K., Winkler M., Racine E., Baquero B., Gittelsohn J. (2020). Healthy food retail during the COVID-19 pandemic: Challenges and future directions. Int. J. Environ. Res. Public Health.

[9] Larson N., Slaughter A.J., Alexander T., Berge J., Harnack L., Neumark-Sztainer D. (2021). Emerging adults’ intersecting experiences of food insecurity, unsafe neighborhoods and discrimination during the coronavirus disease 2019 (COVID-19) outbreak. Public Health Nutr..

[10] Eger L., Komárková L., Egerová D., Mičík M. (2021). The effect of COVID-19 on consumer shopping behaviour: Generational cohort perspective. J. Retail. Consum. Serv..

[11] Bogard J.R., Andrew N.L., Farrell P., Herrero M., Sharp M.K., Tutuo J. (2021). A typology of food environments in the Pacific region and their relationship to diet quality in Solomon Islands. Foods.

[12] Turner C., Aggarwal A., Walls H., Herforth A., Drewnowski A., Coates J., Kalamatianou S., Kadiyala S. (2018). Concepts and critical perspectives for food environment research: A global framework with implications for action in low- and middle-income countries. Glob. Food Sec..

[13] Hu L., Zhao C., Wang M., Su S., Weng M., Wang W. (2020). Dynamic healthy food accessibility in a rapidly urbanizing metropolitan area: Socioeconomic inequality and relative contribution of local factors. Cities.

[14] Glanz K., Salis J.F., Saelen B.E., Frank L.D. (2005). Healthy nutrition environments: Concepts and measures. Am. J. Health Promot..

[15] Lytle L.A., Sokol R.L. (2017). Measures of the food environment: A systematic review of the field, 2007–2015. Health Place.

[16] Gibson D.M. (2011). The neighborhood food environment and adult weight status: Estimates from longitudinal data. Am. J. Public Health.

[17] Cerin E., Frank L.D., Sallis J.F., Saelens B.E., Conway T.L., Chapman J.E., Glanz K. (2011). From neighborhood design and food options to residents’ weight status. Appetite.

[18] Dunn R.A., Sharkey J.R., Horel S. (2012). The effect of fast food availability on fast-food consumption and obesity among rural residents: An analysis by race/ethnicity. Econ. Hum. Biol..

[19] Kolak M., Bradley M., Block D.R., Pool L., Garg G., Toman C.K., Boatright K., Lipiszko D., Koschinsky J., Kershaw K. (2018). Urban foodscape trends: Disparities in healthy food access in Chicago, 2007–2014. Health Place.

[20] Gregson J. (2011). Poverty, Sprawl, and Restaurant Types Influence Body Mass Index of Residents in California Counties. Public Health Rep..

[21] Howard P.H., Fitzpatrick M., Fulfrost B. (2011). Proximity of food retailers to schools and rates of overweight ninth grade students: An ecological study in California. BMC Public Health.

[22] Powell L.M., Bao Y. (2009). Food prices, access to food outlets and child weight. Econ. Hum. Biol..

[23] Black J.L., Macinko J., Dixon L.B., Fryer J.G.E. (2010). Neighborhoods and obesity in New York City. Health Place.

[24] Salois M.J. (2012). Obesity and diabetes, the built environment, and the ‘local’ food economy in the United States, 2007. Econ. Hum. Biol..

[25] Gamba R.J., Schuchter J., Rutt C., Seto E.Y.W. (2014). Measuring the Food Environment and its Effects on Obesity in the United States: A Systematic Review of Methods and Results. J. Community Health.

[26] Xu H.W., Short S.E., Liu T. (2013). Dynamic relations between fast-food restaurant and body weight status: A longitudinal and multilevel analysis of Chinese adults. J. Epidemiol. Community Health.

[27] Ntarladima A., Karssenberg D., Poelman M., Grobbee D.E., Lu M., Schmitz O., Strak M., Janssen N., Hoek G., Vaartjes I. (2022). Associations between the fast-food environment and diabetes prevalence in the Netherlands: A cross-sectional study. Lancet Planet Health.

[28] Morland K.B., Evenson K.R. (2009). Obesity prevalence and the local food environment. Health Place.

[29] Granheim S.I., Løvhaug A.L., Terragni L., Torheim L.E., Thurston M. (2021). Mapping the digital food environment: A systematic scoping review. Obes. Rev..

[30] Brandt E.J., Silvestri D.M., Mande J.R., Holland M.L., Ross J.S. (2019). Availability of Grocery Delivery to Food Deserts in States Participating in the Online Purchase Pilot. JAMA Netw. Open.

[31] Garaus M., Wagner U., Manzinger S. (2017). Happy grocery shopper: The creation of positive emotions through affective digital signage content. Technol. Forecast. Soc. Chang..

[32] Shi K., Shao R., De Vos J., Witlox F. (2021). Do e-shopping attitudes mediate the effect of the built environment on online shopping frequency of e-shoppers?. Int. J. Sustain. Transp..

[33] Huang Y.R., Huang D.N., Nguyen Q.C. (2019). Census tract food Tweets and chronic disease outcomes in the US, 2015–2018. Int. J. Environ. Res. Public Health.

[34] Zhou P.L., Li R.F., Liu K. (2021). The neighborhood food environment and the onset of childhood obesity: A retrospective time-trend study in a mid-sized city in China. Front. Public Health.

[35] Maimaiti M., Zhao X., Jia M., Ru Y., Zhu S. (2018). How we eat determines what we become: Opportunities and challenges brought by food delivery industry in a changing world in China. Eur. J. Clin. Nutr..

[36] Yan S.J., Qu X.T., Lan Y.Y. Research on the Impact of New Retail on Consumer Behavior. Proceedings of the 2018 2nd International Conference on Advances in Management Science And Engineering (AMSE 2018).

[37] Lu M.J., Wang R., Li P.Y. (2021). Comparative analysis of online fresh food shopping behavior during normal and COVID-19 crisis periods. Br. Food J..

[38] Laguna L., Fiszman S., Puerta P., Chaya C., Tárrega A. (2020). The impact of COVID-19 lockdown on food priorities. Results from a preliminary study using social media and an online survey with Spanish consumers. Food Qual. Prefer..

[39] Chen J., Zhang Y., Zhu S., Liu L. (2021). Does COVID-19 Affect the Behavior of Buying Fresh Food? Evidence from Wuhan, China. Int. J. Environ. Res. Public Health.

[40] Shi K., De Vos J., Yang Y., Witlox F. (2019). Does e-shopping replace shopping trips? Empirical evidence from Chengdu, China. Transp. Res. Part A Policy Pract..

[41] Cao X. (2012). The relationships between e-shopping and store shopping in the shopping process of search goods. Transp. Res. Part A.

[42] Pitt E., Gallegos D., Comans T., Cameron C., Thornton L. (2017). Exploring the influence of local food environments on food behaviours: A systematic review of qualitative literature. Public Health Nutr..

[43] Karpyn A., Young C.R., Collier Z., Glanz K. (2020). Correlates of Healthy Eating in Urban Food Desert Communities. Int. J. Environ. Res. Public Health.

[44] Fan S., Teng P., Chew P., Smith G., Copeland L. (2021). Food system resilience and COVID-19—Lessons from the Asian experience. Glob. Food Secur..

[45] Chang M., Green L., Cummins S. (2021). All change. Has COVID-19 transformed the way we need to plan for a healthier and more equitable food environment?. Urban Des. Int..

